# Effects of Berberine on Amelioration of Hyperglycemia and Oxidative Stress in High Glucose and High Fat Diet-Induced Diabetic Hamsters In Vivo

**DOI:** 10.1155/2015/313808

**Published:** 2015-02-01

**Authors:** Cong Liu, Zhuo Wang, Yulong Song, Dan Wu, Xuan Zheng, Ping Li, Jin Jin, Nannan Xu, Ling Li

**Affiliations:** ^1^Department of Endocrinology, Affiliated Shengjing Hospital of China Medical University, Shenyang 110004, China; ^2^Department of Chemistry, Capital Normal University, Beijing 100037, China; ^3^Department of Anesthesiology, Shaanxi Provincial People's Hospital, Xi'an 710068, China

## Abstract

This study investigated the effects of berberine on amelioration of hyperglycemia and hyperlipidemia and the mechanism involved in high glucose and high fat diet-induced diabetic hamsters. Golden hamsters fed with high glucose and high fat diet were medicated with metformin, simvastatin, and low or high dose of berberine (50 and 100 mg·kg^−1^) for 6 weeks. The results showed that the body weights were significantly lower in berberine-treated groups than control group. Histological analyses revealed that the treatment of berberine inhibited hepatic fat accumulation. Berberine significantly reduced plasma total cholesterol, triglyceride, free fatty acid, low density lipoprotein cholesterol, malondialdehyde, thiobarbituric acid-reactive substance, and 8-isoprostane level but significantly increased plasma superoxide dismutase activity. Glucose and insulin levels were significantly reduced in metformin and berberine-treated groups. Glucose tolerance tests documented that berberine-treated mice were more glucose tolerant. Berberine treatment increased expression of skeletal muscle glucose transporter 4 mRNA and significantly decreased liver low density lipoprotein receptor mRNA expression. The study suggested that berberine was effective in lowering blood glucose and lipids levels, reducing the body weight, and alleviating the oxidative stress in diabetic hamsters, which might be beneficial in reducing the cardiovascular risk factors in diabetes.

## 1. Introduction

Glucose disturbance and dislipidemia are often closely related in clinic, and the patients with diabetes are prone to display a profile of dislipidemia. Both hyperglycemia and hyperlipidemia are independent risk factors that work alone or together in accelerating atherosclerosis and diabetic complications [1]. One of the most explored hypotheses that initiate and accelerate atherosclerosis in diabetes is a hyperglycemia- and hyperlipidemia-induced increase in oxidative stress. Oxidative stress is known to be the mechanisms that cause molecular and cellular tissue damage in a wide range of human diseases, including diabetes [2–4]. In fact, oxidative stress is supposed to be associated with the activation of signaling pathways and protein and lipid modification, thus resulting in complications in diabetes [5–7]. Although several lines of medications, including some antidiabetic drugs, declared their effects on reducing the oxidative stress, the results are yet far to be concluded [8–10]. And the challenge of lowering oxidative stress in diabetes has long way to go.

Berberine is a medically important isoquinoline alkaloid extracted from* Coptis chinensis* Franch., which is a kind of antidiarrhea drugs, displaying a broad array of pharmacological effects for more than 1400 years in traditional Chinese medical history [11]. Recently, this natural compound has been increasingly studied for its benefits against various metabolic diseases including diabetes and hyperlipidemia [12, 13].

It has been found that berberine affects glucose metabolism by improving insulin action, increasing insulin secretion, suppressing adipogenesis, and inhibiting mitochondrial function [14]. However, few researches investigated the effects of berberine on oxidative stress. In the present study, we detected the antiglycemic and antilipidemic effects of berberine by measuring blood glucose, lipid profiles, and mRNA expressions of GLUT4 and LDLR in the skeletal muscles or liver of diabetic hamsters. We also investigated whether berberine can ameliorate the oxidative stress, aiming to better understand the mechanism through which berberine improves glucose and lipid metabolism in hyperglycemic and hyperlipidemic hamsters.

## 2. Materials and Methods

### 2.1. Animals and Study Design

Golden hamster, 8 weeks old, weighing 50–70 g (Charles River Laboratories, Vital River, Beijing, China), were housed individually in cages in a temperature controlled room with a 12-hour light/dark cycle. Water was given ad libitum throughout the experiment. The hamsters were put on 8 weeks of high glucose and high fat diet (32% safflower oil, 33.1% casein, 17.6% sucrose, and 5.6% cellulose). High glucose high fat diet (HGHFD) was provided by Beijing Ke Ao Xie Li Feeds Co.

The animals with significantly higher levels of TC and/or TG were fasted for 12 h. The blood samples were obtained from the tail vein of each animal to determine the glucose levels. Fifty animals fed with HGHFD were randomly allocated into five groups (each contained 10 animals): one model control group (MC group) and four treated groups: 50 mg·kg^−1^ metformin (MET: Sino American Shanghai Squibb Pharmaceuticals Co., Ltd., China), 2.0 mg·kg^−1^ simvastatin (SIM: Hangzhou MSD Pharmaceutical Co., Ltd., China), and 50 (low) and 100 (high) mg·kg^−1^ BBR (berberine chloride (C_20_H_18_ClNO_4_), Sigma Chemicals, St. Louis, MO, USA) group. The treatment lasted for 6 weeks.

The animal usage and experimental protocols were approved by China Medical University Animal Care and Use Committee and the Joint Animal Care and Research Ethics Committee of the National Research Council, China. The study was conducted in accordance with the guidelines of the Chinese Council on Animal Care.

### 2.2. Intraperitoneal Glucose Tolerance Test (ipGTT) and Intraperitoneal Insulin Tolerance Test (ipITT)

To determine the effects of berberine on plasma glucose and insulin secretion following the glucose load, ipGTT was performed according to the previously described procedures [15] with slight modifications. In brief, the animals were fasted overnight for 12 h before the last medication and intraperitoneal injection of 50% glucose (1.0 g/kg body weight) was administered. Blood samples were collected before and 15, 30, 60, 90, and 120 minutes after glucose administration with tail vein blood to determine the plasma glucose levels. Insulin levels were measured before the glucose load. The animals were injected with human regular insulin (0.75 U/kg of body weight) into the intraperitoneal space, and blood glucose was assayed immediately before and at 20, 40, 60, and 80 minutes after injection to determine the insulin sensitivity.

### 2.3. Biochemical Analyses

Plasma glucose was measured with glucose oxygenase method (BIOSEN5030, Germany). Plasma insulin levels were determined by radioimmunoassay (Beijing Furui Biological Engineering Co., China). The coefficients of variation were <8% and 10.5%, respectively. Plasma TG, T-Chol, LDL-Chol, and HDL-Chol were measured with enzymatic methods by Abbott Aeroset Chemistry Analyzer (Abbott Laboratories, Abbott Park, IL). Malondialdehyde (MDA), free fatty acid (FFA), and the activity of superoxide dismutase (SOD) levels were determined using detection kits supplied by the Institute of Nan Jing Jian Cheng Bioengineering (Nanjing, China). Malondialdehyde and 8-isoprostane were measured with an ELISA kit (R&D Systems, Minneapolis, MN), a TBARS assay kit (Cayman Chemical Company, Ann Arbor, MI), and an 8-isoprostane enzyme immunoassay kit (Cayman Chemical Company), respectively.

### 2.4. Histological Analysis

Livers were removed and fixed with 10% formalin and embedded in paraffin. Tissue sections were stained with 0.1% (w/v) Oil Red O. There were 10 animals in each group, and, for each animal, 5 pathologic slices from the histological specimens were observed. The pictures we showed were the typical ones in each group.

### 2.5. RT-PCR

The effects of BBR on mRNA level of glucose transporter 4 (GLUT4) and low density lipoprotein receptor (LDLR) were determined by semiquantitation polymerase chain reaction (RT-PCR) in hamsters. RT-PCR was performed as previously described [12, 13]. Total RNA was isolated from the liver and skeletal muscle using TRIzol (Sigma), and cDNA was synthesized with the PrimeScript RT Reagent Kit and gDNA Eraser (Perfect Real Time; Takara, Shiga, Japan). KOD Dash (Toyobo, Osaka, Japan) was used for RT-PCR amplification. The value of gene expression was calculated after normalization to *β*-actin. The primers used in the experiment are listed in the Supplemental Material (available online at http://dx.doi.org/10.1155/2015/313808).

### 2.6. Statistical Analysis

All data were presented as mean values with their standard errors. Significance between groups was analyzed by one-way analysis of variance with GraphPad Prism 4.0 software (San Diego, CA). The statistical significance of differences was assessed with one-way ANOVA.

## 3. Results

### 3.1. Influence of BBR on the Weights of Body and Liver in Hyperglycemic and Hyperlipidemic Hamsters

**Figure 1:**
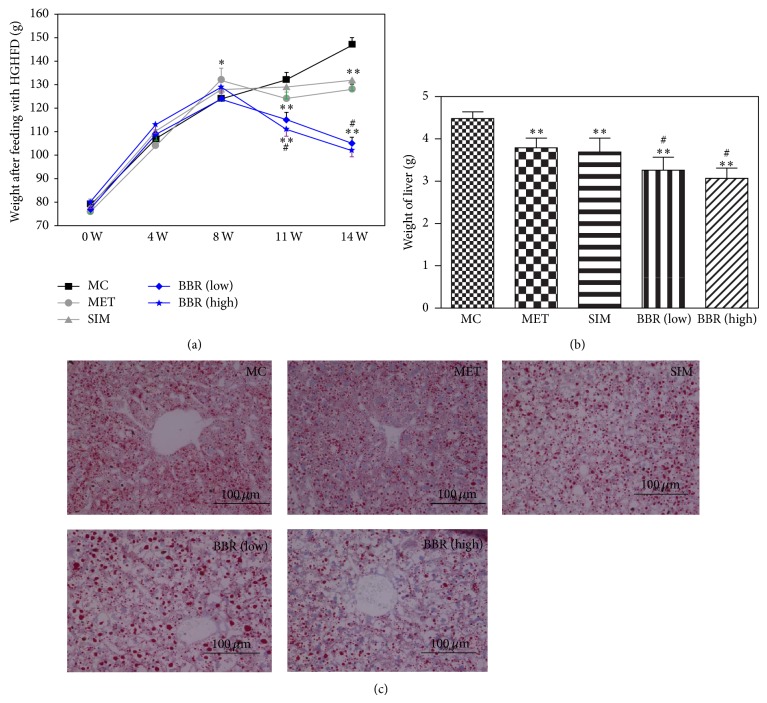
Influence of BBR on the weights of body and liver in diet-induced diabetic and hyperlipidemic hamsters. (a) The weights of diabetic and hyperlipidemic hamsters after medication. (b) Effects of BBR on the liver weight in the diabetic and hyperlipidemic hamsters. (c) Histological findings of the liver stained with Oil Red O. The paraffin slice and Oil Red O staining of hamsters' liver tissues (×200). MC: model control, *n* = 10; MET: metformin, *n* = 10; SIM: simvastatin, *n* = 10; BBR (low): low dose of BBR, *n* = 10; BBR (high): high dose of BBR, *n* = 10. Data are presented as means ± SE. MET, SIM, BBR (low) and BBR (high) Versus MC: ^*^ *P* < 0.05, ^**^ *P* < 0.01; versus MET: ^#^ *P* < 0.05. One-way ANOVA.

The body weights of hyperglycemic and hyperlipidemic hamsters were measured after HGHFD for 8 weeks (Figure 1(a)). After six weeks of medication, the body weights in both low and high doses of BBR-treated groups were significantly lower than those of MC (*P* < 0.01) group.

After high fat diet, the weights of liver in the hamsters of MET-, SIM-, and BBR- (low and high dose) treated groups reduced significantly compared with those of MC (Figure 1(b): *P* < 0.01). Histological analyses revealed that the treatment with BBR inhibited hepatic fat accumulation, while abundant lipid droplets were present in the livers of high fat diet-fed hamsters (Figure 1(c)).

### 3.2. Effects of BBR on the Glucose Metabolism in Hamsters

**Figure 2:**
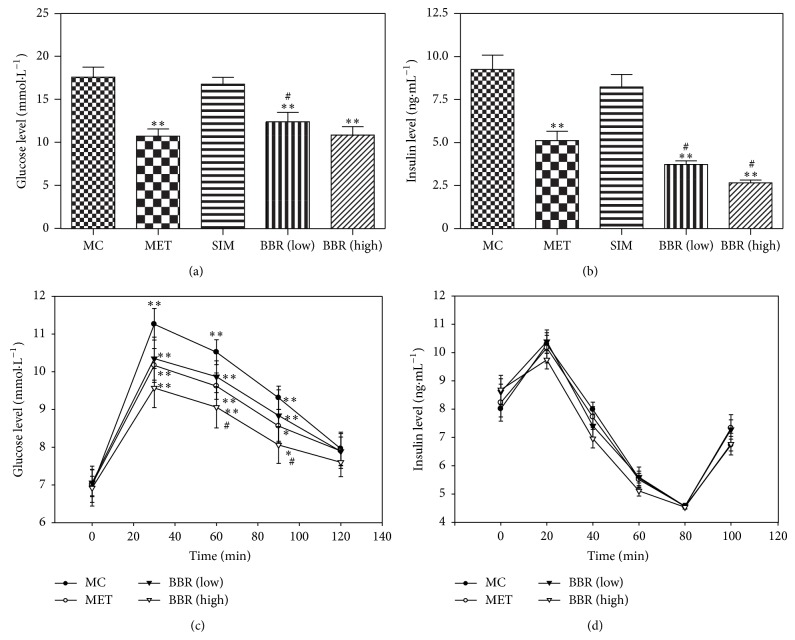
Effects of BBR on the glycometabolism in hamsters. The glucose levels (a), insulin levels (b), the glucose tolerant test (ipGTT) (c), and insulin tolerant test (d) were carried out in the control hamsters after medication with MET, SIM, or BBR. MC: model control, *n* = 10; MET: metformin, *n* = 10; SIM: simvastatin, *n* = 10; BBR (low): low dose of BBR, *n* = 10; BBR (high): high dose of BBR, *n* = 10. Data are presented as means ± SE. MET, SIM, BBR (low) and BBR (high) Versus MC: ^*^ *P* < 0.05, ^**^ *P* < 0.01; versus MET: ^#^ *P* < 0.05. One-way ANOVA.

The blood glucose level reduced significantly in hyperglycemic and hyperlipidemic hamsters after medicating with MET and low and high levels of BBR (Figure 2(a): *P* < 0.01), while medication with SIM did not influence the level of blood glucose of the animals.

Insulin levels were significantly reduced in both MET- and BBR-treated groups following the high fat diet (Figure 2(b): *P* < 0.01).

In ipGTT, the blood glucose levels increased significantly 30 min after injection with glucose, then reduced gradually, and resumed to the normal level at 120 min. The blood glucose levels in the MET group and low or high dose of BBR groups were lower than those in MC group (Figure 2(c): *P* < 0.01; *P* < 0.05).

By contrast, the insulin sensitivity did not change significantly following the treatment with MET and low or high dose of BBR (Figure 2(d)), indicating that, following the BBR treatment, these hyperglycemic and hyperlipidemic animals became better tolerant to the glucose load but had no significant change in insulin sensitivity.

### 3.3. BBR Changed Lipid Profile and Reduced Oxidative Stress Related to Hyperglycemic and Hyperlipidemic Hamsters

**Figure 3:**
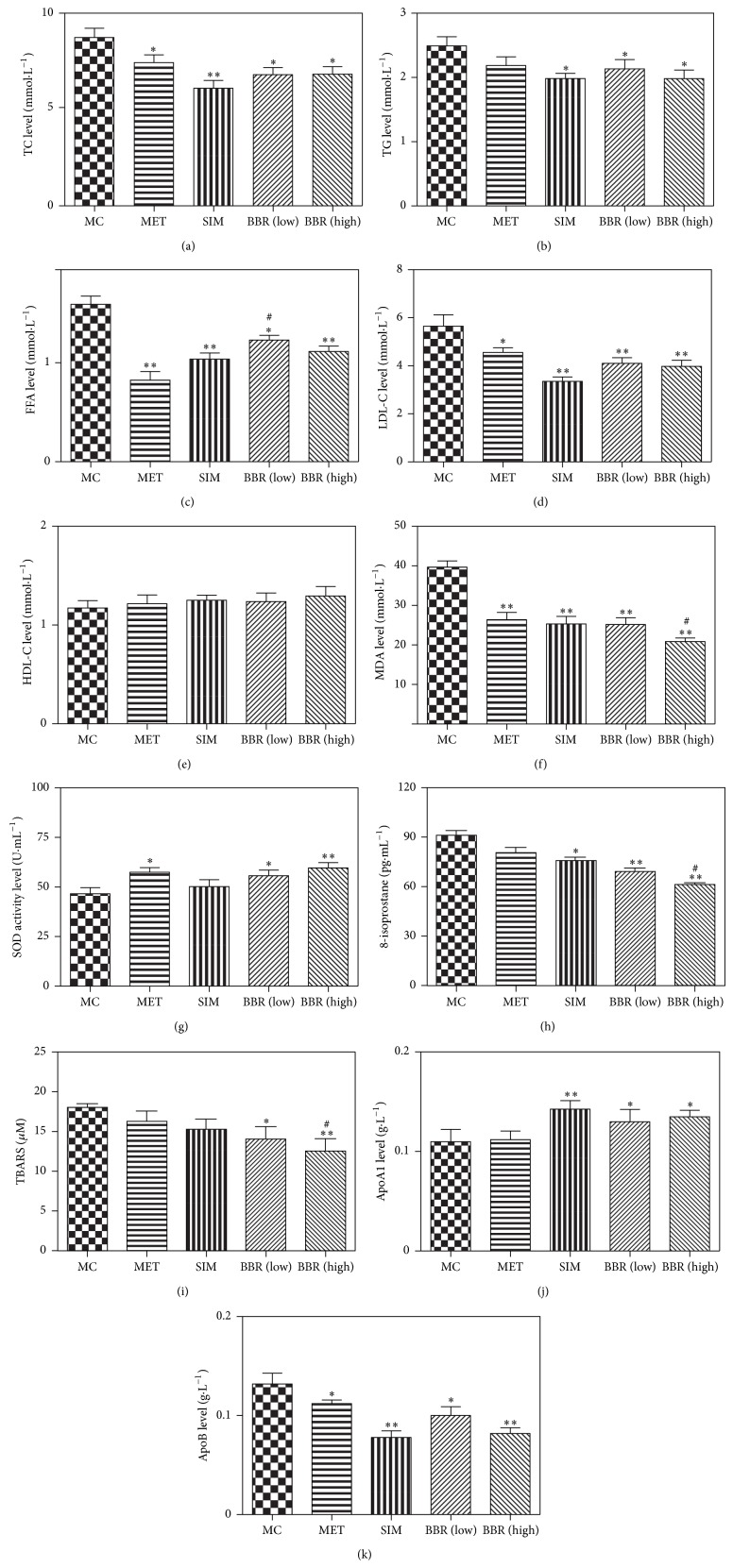
Effects of BBR on regulating the lipid-related biomarker of blood TC ((a) total cholesterol), TG ((b) triglyceride), FFA ((c) free fatty acid), LDL ((d) low-density lipoprotein cholesterol), HDL-C ((e) high-density lipoprotein cholesterol), MDA (f), SOD (g), 8-isoprostane (h), TBARS (i), ApoA1 (j), and ApoB (k) in the diet- and STZ-induced diabetic and hyperlipidemic hamsters. Values were calculated according to the levels of blood lipids. MC: model control, *n* = 10; MET: metformin, *n* = 10; SIM: simvastatin, *n* = 10; BBR (low): low dose of BBR, *n* = 10; BBR (high): high dose of BBR, *n* = 10. Data are presented as means ± SE. MET, SIM, BBR (low) and BBR (high) Versus MC: ^*^ *P* < 0.05, ^**^ *P* < 0.01; versus MET: ^#^ *P* < 0.05. One-way ANOVA.

BBR significantly reduced the levels of plasma TC (Figure 3(a): *P* < 0.05; *P* < 0.01), TG (Figure 3(b): *P* < 0.05), FFA (Figure 3(c): *P* < 0.05; *P* < 0.01), and LDL-C (Figure 3(d): *P* < 0.05; *P* < 0.01) but did not influence that of HDL-C in hyperglycemic and hyperlipidemic hamsters (Figure 3(e)).

BBR, MET, and SIM reduced the levels of plasma MDA significantly (Figure 3(f): *P* < 0.01). And BBR and MET increased the plasma SOD activity in hyperglycemic and hyperlipidemic hamsters in a dose-dependent manner (Figure 3(g): *P* < 0.05; *P* < 0.01).

Furthermore, the levels of oxidative stress markers, thiobarbituric acid-reactive substance (TBARS), and 8-isoprostane were significantly lower in BBR than in MC (Figures 3(h)-3(i): *P* < 0.05; *P* < 0.01).

Both BBR and SIM increased the plasma level of ApoA1, but this increase was not significant following the BBR medication (Figure 3(j)). However, both BBR and SIM significantly reduced plasma ApoB level stimulated by hyperglycemia and hyperlipidemia in hamsters (Figure 3(k): *P* < 0.05; *P* < 0.01).

### 3.4. Influence of BBR on GLUT4 and LDLR mRNA in Diabetic and Hyperlipidemic Hamsters

**Figure 4:**
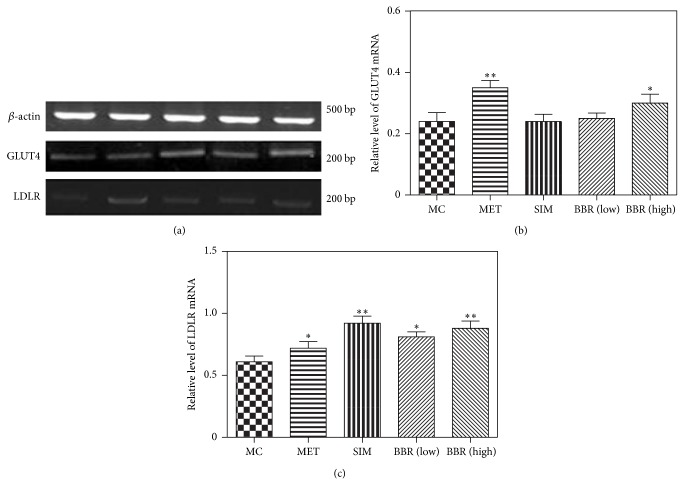
The determination of GLUT4 and LDLR PPAR*γ* mRNA levels in the skeletal muscle or liver of hamsters by RT-PCR. (a) The electrophoretogram of *β*-actin, GLUT4, and LDLR by RT-PCR. (b) Relative level of GLUT4 mRNA in the skeletal muscle of hamsters. (c) Relative level of LDLR mRNA in the liver of hamsters. MC: model control, *n* = 10; MET: metformin, *n* = 10; SIM: simvastatin, *n* = 10; BBR (low): low dose of BBR, *n* = 10; BBR (high): high dose of BBR, *n* = 10. Data are presented as means ± SE. MET, SIM, BBR (low) and BBR (high) Versus MC: ^*^ *P* < 0.05, ^**^ *P* < 0.01. One-way ANOVA.

The expression of muscle GLUT4 mRNA was enhanced significantly by either high dose of BBR or MET medication (Figures 4(a) and 4(b): *P* < 0.05; *P* < 0.01).

The LDLR mRNA expression of liver was significantly increased by the treatment of SIM and low or high dose of BBR (Figures 4(a) and 4(c): *P* < 0.05; *P* < 0.01).

## 4. Discussion

Berberine is a widely used drug in China. It is commonly used as an anti-inflammatory traditional medicine. Berberine is from a group of isoquinoline alkaloids, which is found in the roots, rhizomes, and stem bark of various plants including* Coptis chinensis* (Huanglian), Rhizoma Coptidis, and* Hydrastis canadensis*. It is a traditional Chinese herb and commonly used in the treatment of diarrhea [16]. Many studies have suggested that berberine has beneficial effects on diabetes and hyperlipidemia, yet little is known regarding the efficacy of berberine on oxidative stress in diabetes.

The present study detected the effect of berberine on the glucose and insulin secretion in diabetes and the results showed that berberine effectively reduced the glucose level and body weight in diabetic hamsters. And the insulin secretion was also reduced by high dose of berberine treatment.

Since berberine was observed to act as an insulin-sensitizing agent in cultured cells, its activity was often compared with that of metformin, but the results were controversial l [17–19]. Here we also compared the efficacy of berberine with that of metformin; the results showed that glucose-lowering effect of berberine was less than that of metformin but with a better body weight reduction and decreased insulin and oxidative stress levels than metformin. It has been reported that berberine ameliorates hyperglycemia by enhancing insulin secretion, stimulating glycolysis, suppressing adipogenesis, and inhibiting mitochondrial function [14]. Here we showed that berberine improved the glucose metabolism by increasing glucose transporter 4 (GLUT4) in skeletal muscles. The GLUT4 mRNA expression, which was reduced significantly in the skeletal muscles of diabetic and hyperlipidemic hamsters in our study, was upregulated by the treatment with berberine, suggesting that berberine improved the glucose control by elevating the glucose availability in peripheral tissues through GLUT4 in skeletal muscles.

As regards lipid metabolism, the lipid-lowering effect of berberine has already been suggested in animals and also human by some small clinical trials. And the antihyperlipidemia effects of berberine appear to be related to stabilization of hepatic LDL-R [20]. It is proposed that the pathogenesis of hypercholesterolemia is involved in a number or abnormalities, including excessive production of LDLR [21]. Promoting the expression of LDLR in the liver plays a pivotal role in the homeostasis of endogenous cholesterol and LDLR is also one of the key factors in regulating plasma ApoB [22, 23]. Recent studies showed that berberine exerted the effects of reducing blood lipids by controlling the expression of LDLR mRNA after transcription and by increasing the stability of LDLR mRNA [24]. The present study showed that berberine reduced the plasma levels of LDL and ApoB, inhibiting hepatic fat accumulation, and promoted the expression of liver LDLR mRNA significantly in hamsters with diabetes and hyperglycemia, confirming that berberine exerted the lipid-lowing effect by enhancing the expression of LDLR mRNA.

Oxidative stress is known to be increased in diabetes, since increased glucose both enhances oxidant production and impairs antioxidant defenses by multiple mechanisms, including increased intracellular generation of reactive oxygen species (ROS) [25]. It is believed that malondialdehyde (MDA) is one of oxygenated aldehydes compounds that produced during the formation and attack of free radicals to tissues [26, 27]. Enzymic superoxide dismutase (SOD) might alleviate the damage. And oxidative stress can lead to endothelial cell dysfunction and vascular smooth muscle cell damage as well as abnormal glucose metabolisms in diabetes; the latter in turn worsen the oxidative stress. In accordance with those studies, the high fat feeding of hamsters in the present study resulted in the reduction of SOD and enhancement of MDA, indicating that oxidative stress could be induced by high glucose high fat consumption. It is proposed that SOD plays an important role in regulating the oxidative and antioxidative homeostasis in rodent by clearing superoxide anion free radicals O_2_^−^ and protecting the cells from damage [28].^.^ Here we showed that berberine not only improved lipid profiles but reduced the plasma MDA level and increased the SOD activity, TBARS, and 8-isoprostane significantly, indicating that berberine possessed the antioxidative effects on protecting against the oxidative stress-related damage in hamsters following the high glucose high fat load.

Diabetes mellitus is associated with high risk of cardiovascular events and excess cardiovascular mortality, because hyperglycemia and hyperlipidemia are both the initial events in triggering the oxidative stress which can accelerate the development of atherosclerosis and progression of complications in diabetes [29].

Lowering the risk factors of cardiovascular events in diabetes has always been the focus of diabetic treatment clinically. Two clinical trials, The Diabetes Control and Complications Trial (DCCT) and The UK Prospective Diabetes Study (UKPDS), have shown that the management of glucose and lipids would reduce the complications of diabetes [30–33].

In the present study, we demonstrated that berberine effectively achieved blood glucose and lipids control and reduced body weight as well as alleviating oxidative stress in diabetic hamsters, which yield potential benefits on the cardiovascular risks in diabetes. We therefore postulated that berberine might have the potential in reducing the cardiovascular disease in diabetes. More evidence is needed to support our conclusions.

## Supplementary Material

The primers used in the RT-PCR experiment are listed in Table 1.
