# Supplementary material for: Effects of Berberine on Amelioration of Hyperglycemia and Oxidative Stress in High Glucose and High Fat Diet-Induced Diabetic Hamsters In Vivo
Source: Biomed Res Int. 2015 Feb 1;2015:313808. doi: 10.1155/2015/313808 (PMC4331319; doi:10.1155/2015/313808)
Supplement: Supplementary file 1 — The primers used in the RT-PCR experiment are listed in Table 1. [file 313808.f1.pptx]

## Slide 1
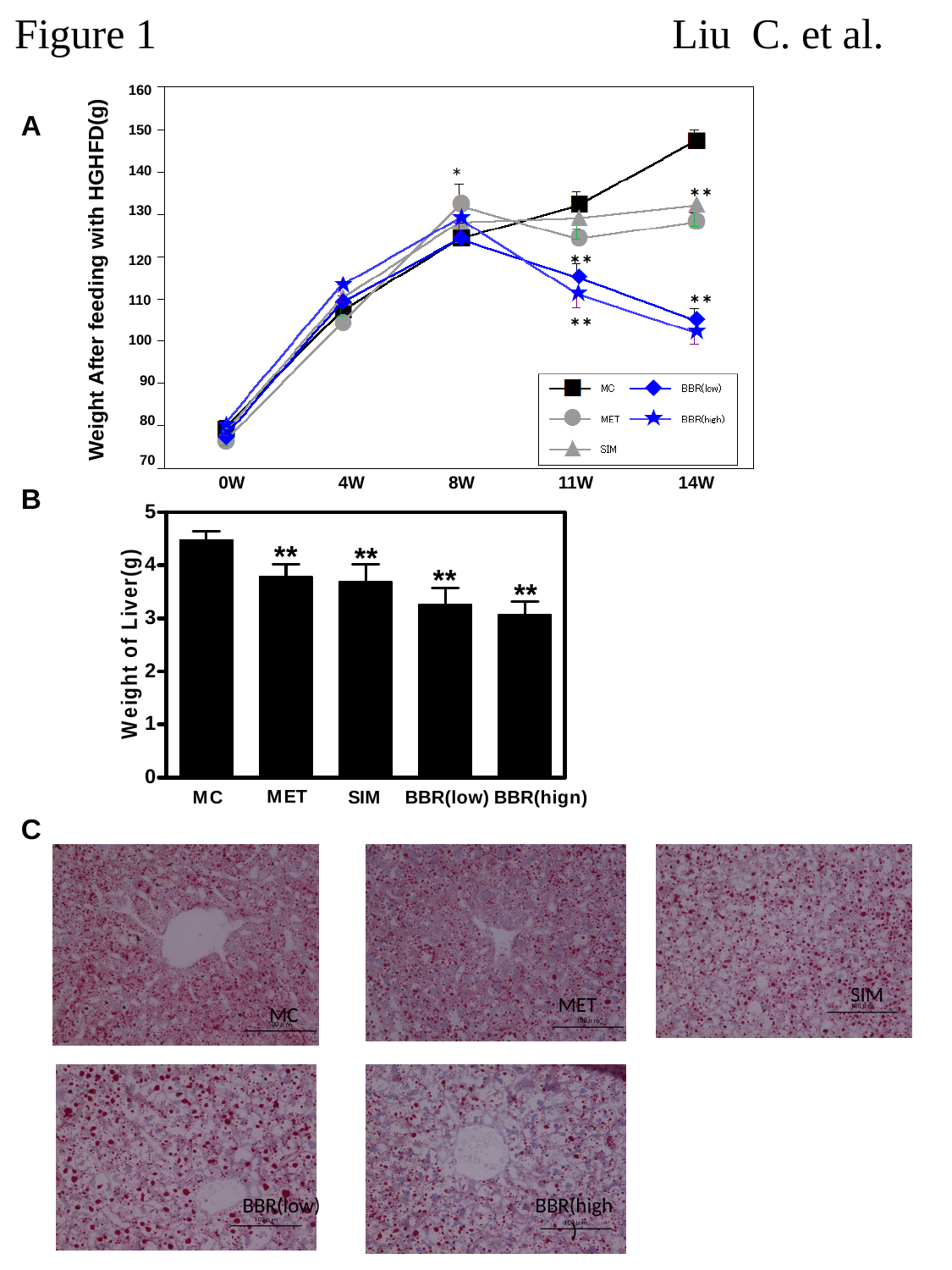

Figure 1
Liu C. et al.
160
A
150
140
*
**
130
**
120
Weight After feeding with HGHFD(g)
**
110
**
100
90
80
70
0W
4W
8W
11W
14W
B
C
SIM
MET
MC
BBR(low)
BBR(high)

## Slide 2
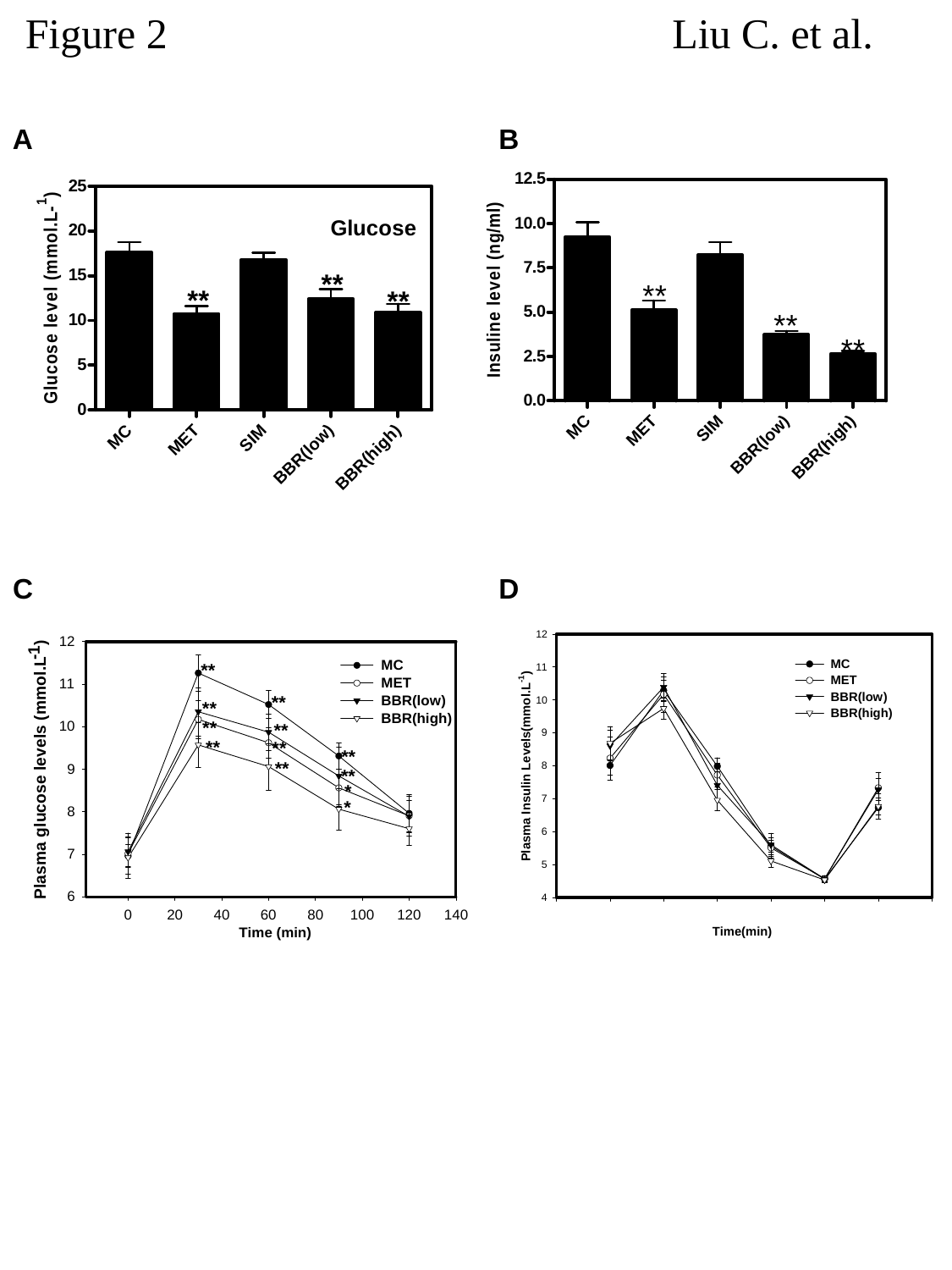

Figure 2
Liu C. et al.
A
B
C
D

## Slide 3
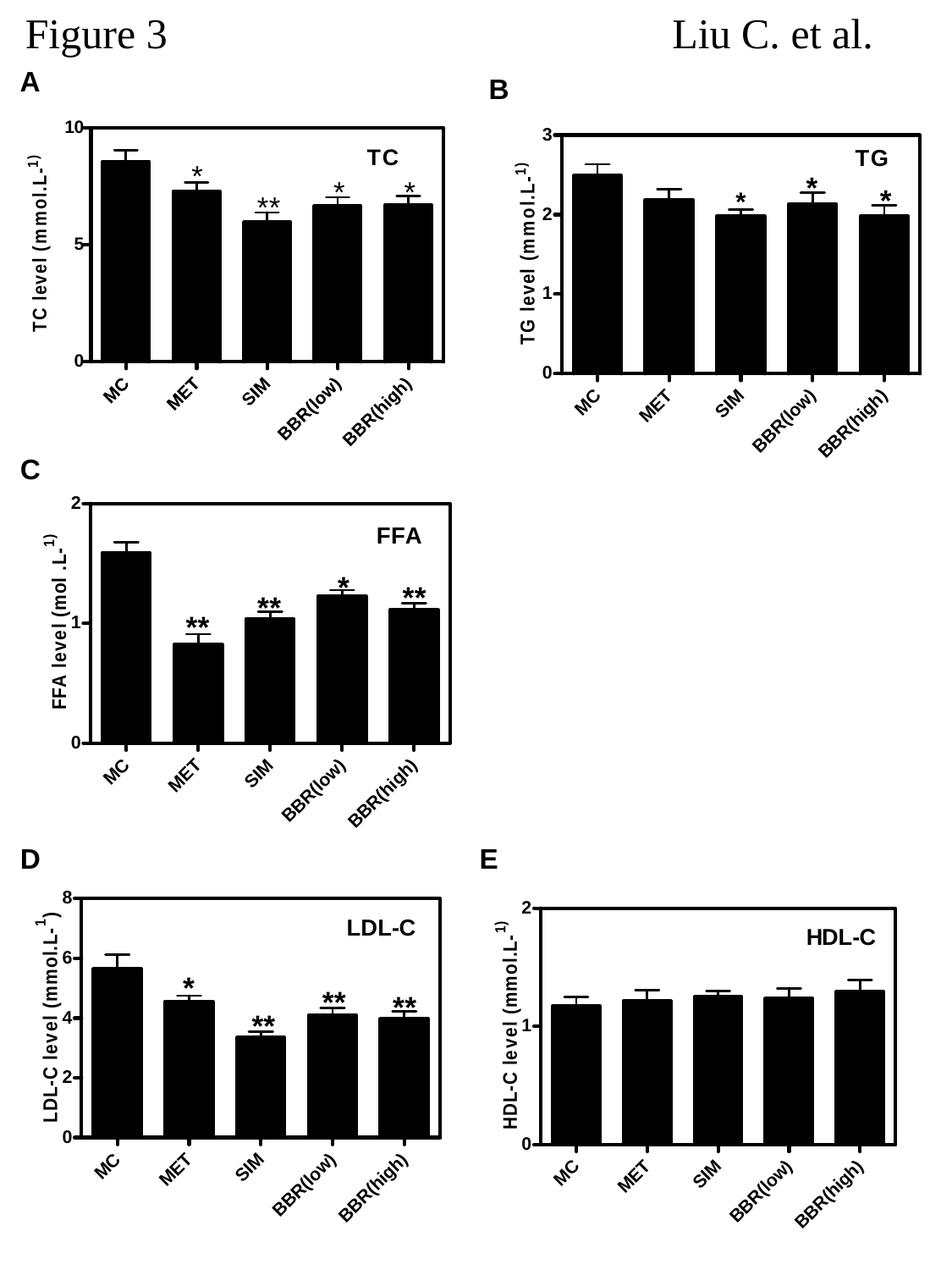

Figure 3
Liu C. et al.
A
B
C
D
E

## Slide 4
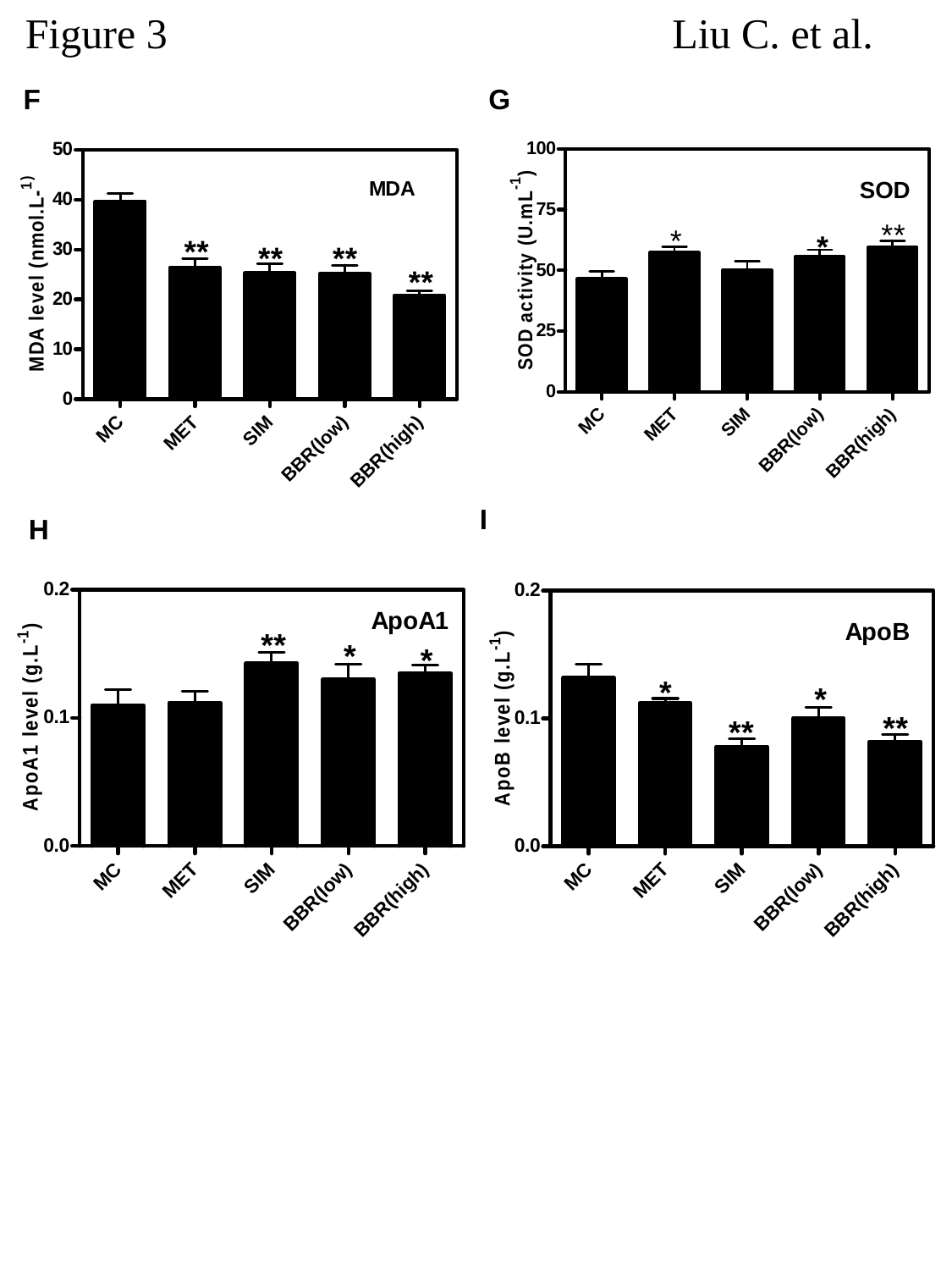

Figure 3
Liu C. et al.
F
G
I
H

## Slide 5
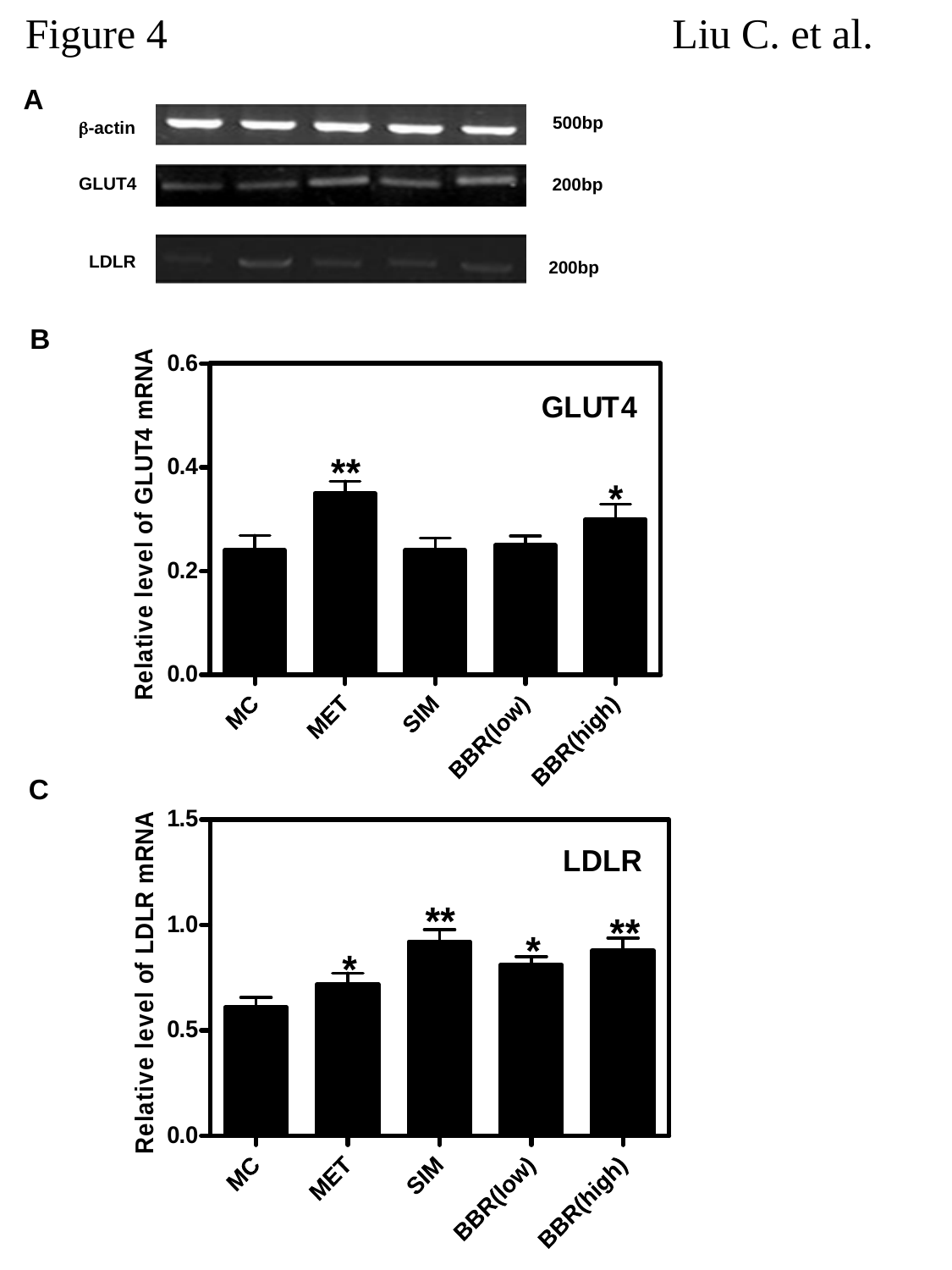

Figure 4
Liu C. et al.
A
500bp
β-actin
GLUT4
200bp
LDLR
200bp
B
C
